# Association of dietary patterns with continuous metabolic syndrome in children and adolescents; a nationwide propensity score-matched analysis: the CASPIAN-V study

**DOI:** 10.1186/s13098-018-0352-3

**Published:** 2018-07-03

**Authors:** Roya Kelishadi, Ramin Heshmat, Marjan Mansourian, Mohammad Esmaeil Motlagh, Hasan Ziaodini, Majzoubeh Taheri, Zeinab Ahadi, Tahereh Aminaee, Azam Goodarzi, Morteza Mansourian, Mostafa Qorbani, Nafiseh Mozafarian

**Affiliations:** 1 Child Department of Pediatrics, Child Growth and Development Research Center, Research Institute for Primordial Prevention of Non-communicable Disease, Isfahan University of Medical Sciences, Isfahan, Iran; 2 Chronic Diseases Research Center, Endocrinology and Metabolism Population Sciences Institute, Tehran University of Medical Sciences, Tehran, Iran; 3 School of Health, Isfahan University of Medical Sciences, Isfahan, Iran; 4 Department of Pediatrics, Ahvaz Jundishapur University of Medical Sciences, Ahvaz, Iran; 5 Office of Health and Fitness, Ministry of Education, Tehran, Iran; 6 Bureau of Population, Family and School Health, Ministry of Health and Medical Education, Tehran, Iran; 7 Department of Health Education and Promotion, Tarbiat Modarres University, Tehran, Iran; 8 Health Management and Economics Research Center, Iran University of Medical Sciences, Tehran, Iran; 9 Non-communicable Diseases Research Center, Alborz University of Medical Sciences, Karaj, Iran; 10 Endocrinology and Metabolism Research Center, Endocrinology and Metabolism Clinical Sciences Institute, Tehran University of Medical Sciences, Tehran, Iran

**Keywords:** Metabolic syndrome, Dietary pattern, Propensity score, Children

## Abstract

**Objective:**

This study aims to determine the association of dietary patterns with metabolic syndrome (MetS) and its components in children and adolescents.

**Methods:**

This nationwide study was conducted in 2015 among 4200 students aged 7–18 years, who lived in 30 provinces in Iran. The analysis was conducted based on the propensity score using a matched case–control study design. Three dietary patterns were obtained conducting a principal component analysis with a varimax rotation on 16 dietary groups. Continuous MetS score was computed by standardizing the residuals (z-scores) of MetS components by regressing them according to age and sex. The gold standard diagnosis of MetS was considered based on the International Diabetes Federation criteria. Moreover, for the purpose of data analysis, matched logistics analysis was used.

**Results:**

The study participants consisted of 3843 children and adolescents (response rate 91.5%) with mean (SD) age of 12.45 (3.04) years. Totally 49.4% of students were girls and 71.4% lived in urban areas. Three dietary patterns were obtained: Healthy, Western, and Sweet. Prevalence of MetS was 5% (boy 5.5 and girl 4.5%). Results of multivariate analysis show that students with Sweet dietary patterns were at higher risk for abdominal obesity (OR 1.29; 95% CI 1.01–1.66), elevated blood pressure (OR 1.35; 95% CI 1.01–1.81) and MetS (OR 1.33; 95% CI 1.02–1.74). The two other dietary patterns were not associated with MetS and its components.

**Conclusion:**

Sweet dietary pattern increase the risk of MetS and some its components in Iranian children and adolescents. This finding provides valuable information for effective preventive strategies of MetS based on diet rather than medication to maintain healthy lifestyle habits.

## Background

Metabolic syndrome (MetS) is a predisposing factor for many chronic diseases and diabetes mellitus type 2, which begins in childhood and reveals itself in adulthood. This syndrome is a set of metabolic abnormalities [1, 2]. Prevalence of MetS in children and adolescents in pace with the increasing prevalence of obesity and overweight is increasing rapidly [3]. Control of MetS in childhood is very important, as this syndrome causes increased risk of cardiovascular diseases in adulthood [4, 5].

Various criteria are used for diagnosis of MetS among children and adolescents and there is no consensus over its definition in the entire world [6]. According to different criteria, the prevalence of MetS varied 2.5- in Iranian children and adolescents [7–9].

Several important factors have been cited for MetS, some of which include genetic, socio-economic and environmental factors, unhealthy eating habits and development of urbanization [10]. However, diet plays a major role in the incidence of MetS [11]. Also, dietary patterns during childhood are reported to remain relatively stable throughout life, and are associated with long-term health, particularly with cardiovascular risk factors in adulthood [12]. Today, as suggested by researchers, in identifying the relation between nutrition and diseases, diets are considered as dietary patterns so that they could be studied in general (rather than as separate components) [13]. Several studies have examined the association between dietary patterns and MetS in adults [14–19]. A number of studies in different countries have studied the relationship between dietary patterns and the risk of MetS or its components in children and adolescents [20–24]. In Iran, some studies have already examined the relationship between dietary patterns with cardiometabolic risk factors [25–27]. However, a thorough investigation of the literature did not yield any research on the relationship between dietary patterns with MetS in this age group, and only a limited number of studies have been performed on adults [28–30].

In recent years, lifestyles of children and adolescents have diversified, and there is a growing tendency among them towards calorie rich foods without adequate nutritional value. Moreover, the prevalence of MetS in adolescents has been observed to be associated with a progressive increase in the prevalence of obesity and overweight. Therefore, this study was designed to investigate whether dietary patterns and MetS are correlated in a national sample of 7–18 year-old Iranian children and adolescents using the propensity score (PS).

## Methods

This study was conducted using a matched case–control study design. The data were collected from the fifth phase of a national project entitled “Childhood and Adolescence Surveillance and PreventIon of Adult Non-communicable disease” (2014–2015). The protocol details of the CASPIAN-V study have been explained in an earlier publication [31].

This national multicenter study was conducted between the year 2014 and 2015 on 7–18 year old students with the cooperation and coordination of the Ministry of Health and Medical Education, Ministry of Education, Child Development Research Center (CDRC) at the Isfahan University of Medical Sciences, and Endocrinology and Metabolism Research Institute (EMRI) at the Tehran University of Medical Sciences. The study was reviewed and confirmed by the Ethics Committee of Isfahan and Tehran University of Medical Sciences. Written and oral consent was obtained from the students and their parents.

A stratified, multi-stage cluster sampling design was used to randomly select students from urban and rural area in 30 provinces of the country. As a result, 48 clusters of 10 people in each province of the country, making a total of 14,400 people were surveyed. From among the 48 clusters in each province, 14 clusters were randomly selected for collection of the required blood sample; therefore, a sample size of 4200 patients was obtained. In this study, all adolescents participating in the CASPIAN study who had complete laboratory data were enrolled.

The data required for the study were collected using a structured questionnaire and via conducting interviews with students eligible to respond in schools, as well as through performing clinical examinations, anthropometric measurements and blood tests. The internal consistency of the questionnaire was approved in the previous CASPIAN studies with the overall Cronbach’s alpha value of 0.93 and range of 0.92–0.97 and also, the reliability was confirmed as 0.94 through conducting test–retest measurement [32].

After identifying the eligible students, parents of the students were invited to complete the questionnaires by coordinating with school officials. To this end, presence of at least one parent was necessary and sufficient, and presence of both parents was not required. The respondents were asked demographic information including age, gender, place of residence, duration of sleep, eating habits, physical activity and time spent watching TV and using computers. Also, a number of questions about socioeconomic status (SES) including parents education, parents occupation, family assets and type of school for students), and family history of chronic disease were asked from the parents.

With regard to the eating habits of the students, 16 dietary groups, including sweets (cakes, cookies, pastries, biscuits and chocolate), salty snacks (snacks, chips and pretzels), carbonated drinks, diet sodas, non-alcoholic beer, fresh fruit, dried fruit, fresh fruit juice, vegetables (fresh or cooked vegetables), packed fruit juice, milk, yogurt, cheese, fast food (sausages, burgers and pizza), tea, and sugar and sugar cube along with tea and coffee were examined. The students were asked to report how many times they used such foods; the responses were ranked based on a 4 point Likert scale with 1 representing “never”, 2 representing “rarely”, 3 representing “weekly” and 4 representing “daily”.

The dietary patterns were obtained conducting a principal component analysis (PCA) with a varimax rotation. Load factor values ≥ 0.2 were considered to determine the dietary groups in each dietary pattern. As a result, three dietary patterns were identified among the study participants: healthy diet, Western dietary pattern and sweet dietary pattern. Then, all the three patterns were grouped into two-state variables (values below the median were codified as 0 and values above the median as 1).

Anthropometric measurements, including measurements of height, weight and waist circumference (WC), were performed by a trained team based on a standard program using validated instruments [32]. Height was measured to the nearest 1 cm with one decimal in millimeters using a non-elastic tape meter while subjects were in a barefoot standing position, with their shoulders in a normal position. Students’ weights with a decimal point were recorded using an exact weight scale, the accuracy of whose weights’ was carefully evaluated by standard weights. Their WC were also measured by a non-elastic cloth meter. WC was measured in the most condensed part at the halfway between the lower rib margin and anterior superior iliac spine while the subjects were at the end of normal exhalation and was recorded to the nearest 0.1 cm. Abdominal obesity was considered as waist to height ratio (WHtR) greater than 0.5 [33].

Blood pressure (BP) was also measured twice in the right arm of subjects who had been resting for at least 5 min in a seated position using a mercury sphygmomanometer. The mean of these two measurements was calculated and considered as an individual’s BP. Mean arterial blood pressure (MAP) was calculated using the equation below:

$${\text{MAP}} = \left[ {\left( {{\text{Systolic}}\;{\text{blood}}\;{\text{pressure}} - {\text{diastolic}}\;{\text{blood}}\;{\text{pressure}}} \right)/3} \right] + {\text{diastolic}}\;{\text{blood}}\;{\text{pressure}}$$

To collect blood samples, students along with one of their parents referred to the laboratory. Then, 0.6 cc of venous blood samples was taken from their anti-cubital vein after 12 h of overnight fasting. Up to 1 cc of blood samples was poured in tubes containing EDTA and rapidly analyzed in terms of the CBC test. The remaining blood sample (5 cc) was placed in a plastic tube and centrifuged. Closed micro tubes were filled up with 200 ml of blood samples and transferred to the coldest freezer. Frozen blood and plasma samples were kept in the freezer at −70 °C and then transferred to the central laboratory in shortest time possible. Laboratory findings included fasting blood sugar (FBG), triglycerides (TG) and high density lipoprotein cholesterol (HDL-C) measured enzymatically using automated analysis.

### Calculation of continuous MetS score

The *cMetS* is calculated by standardizing WC, MAP, HDL-C, TG, and FBG using regressions on age and gender [34]. Since the HDL is reversely related to the risk of MetS, it is multiplied by a negative number. According to the following equation, the *cMetS* is calculated for each individual by summing standard remainders of Z scores. The higher cMetS score revealed more adverse metabolic status.

$${\text{cMetS}}\;{\varvec{score - }} = {\text{zHDL}} + {\text{zFBG}} + {\text{zTG}} + {\text{zWC}} + {\text{zMAP}}$$

Following the calculation of cMetS, the ROC curve analysis (a receiver operating characteristic curve) with an estimate of sensitivity and specificity was used to determine an optimal cMetS cut-off point to predict MetS.

The gold standard for the diagnosis of MetS was considered according to the IDF (International Diabetes Federation) benchmark for possessing at least 3 of the following components: fasting TG ≥ 150 mg/dl; HDL-c ≤ 40 mg/dl; WHtR > 0.5; SBP and/or DBP > 90th percentile for sex, age and height, from national reference cut-off points; and FBG ≥ 100 mg/dl [35]. The best cut-off point of MetS was obtained based on the lowest score [36] with maximum sum of sensitivity and specificity. Concerning the specified cut off point, individuals were divided into two categories: with MetS and without MetS.

### Statistical analysis and matching based on PS

To compare quantitative and qualitative variables, independent sample *t* test and Chi square test were used, respectively. The quantitative variables were reported as mean and standard deviation and qualitative variables were presented as frequencies and percentages.

PS was calculated based on conditional logistic model with potential confounding variables (age, sex, living area, SES, parent perception about student body image, weigh satisfaction, physical activity, sleep duration, family history of chronic diseases, parents’ BMI, screen time). Two groups (with MetS and healthy) were matched based on 1:1 matching method without replacement by the score. Then standardized skewness percentages before and after synchronization were calculated for each variable and then standardized skewness mean was calculated for all variables. After matching, the odds ratio (OR) and 95% confidence interval (CI) was calculated for matched data.

All statistical analysis was performed in Stata 10 software (StataCorp, College Station, Texas, USA) using survey analysis methods. The P-value < 0.05 was considered as statistically significant.

## Results

Of 3755 students aged 7–18 years, there were 1975 (52.6%) boys and 2732 (72.8%) individuals living in urban areas. The mean age of participants was 12.44 (3.04) years. Three dietary patterns were obtained using factor analysis for 16 dietary groups: [1] Healthy food which were rich in fresh fruit, dried fruit, fresh fruit juice, vegetables, milk, yogurt, and cheese; [2] sweet pattern which were rich in sweet snacks (cakes, cookies, pastries, biscuits, chocolate), soft drinks, packaged fruit juice, tea, cubed sugar and sugar; and [3] Western pattern with high consumption of salty snacks (cheese puffs, chips and pretzels), beer, fast foods (sausages, burgers and pizza) and coffee. These three dietary patterns totally explained 38.18% of the consumption variance (Table 1).

**Table 1:** Factor-loading matrix for major dietary patterns

Food groups	Dietary patterns		
	Healthy pattern	Sweet pattern	Western pattern
Sweets^a^		0.451	0.332
Salty snacks^b^			0.449
Soft drinks		0.452	0.352
Diet soda		0.263	
Delester		− 0.323	0.521
Fresh fruits	0.593		
Dried fruits	0.724		
Fresh juices	0.623		
Canned fruit juice	0.309	0.411	0.268
Vegetables	0.473	0.201	
Milk	0.560		
Yogurt	0.582		
Cheese	0.433		− 0.343
Fast foods^c^			0.659
Tea		0.752	− 0.218
Sugar	− 0.205	0.755	− 0.241
Coffee	0.299		0.579

Factor loadings with absolute values < 0.2 are not shown for clarity

^a^ Biscuits, cookies, cakes, chocolates, candies

^b^ Chips, cheese curls, popcorn, pretzels

^c^ Pizza, hamburgers, sausage

Prevalence of MetS according to the IDF criteria was equal to 6.3% with the ratio of 7.5% for boys versus 5% for girls. These ratios in two groups were significantly different (P = 0.002). In this regard, 37.3% of individuals had no factor of MetS components. Of the participants, 37.3, 19.1, 5.5, 0.7, and 0.1% had 1, 2, 3, 4, and 5 factors of MetS components, respectively. Based on the ROC curve on a cMetS, the best cutoff point was 1.9749 with a sensitivity of 95.3% and specificity 85.5% to diagnose individuals with MetS. Furthermore, the area under curve (AUC) and the confidence interval were 0.953 and 0.943–0.962, respectively, indicating high accuracy to determine the status of MetS in individuals. According to the cut-off point, the incidence of MetS was 19.6 percent (735 patients).

**Fig. 1:**
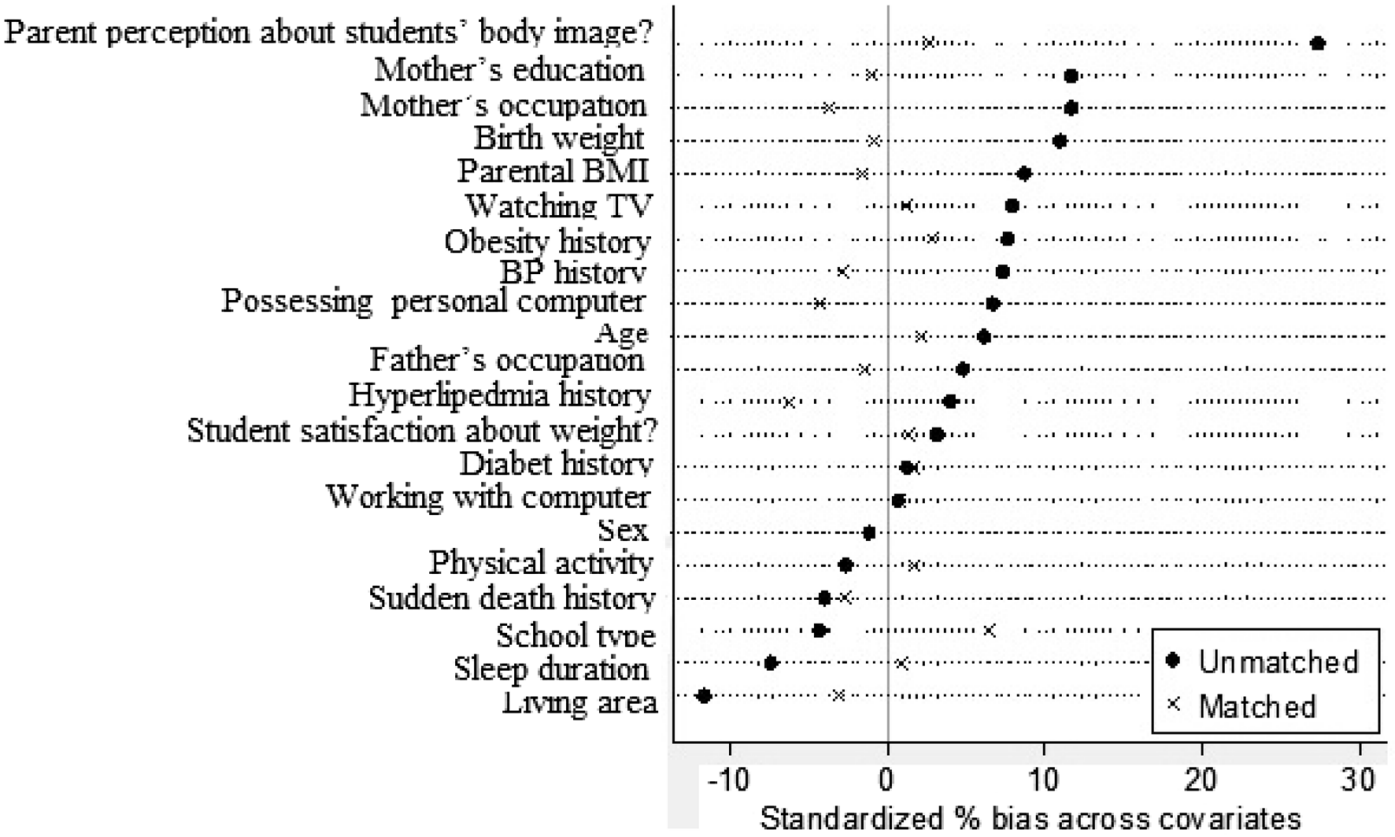
Standardized differences for baseline covariates in the original and the matched sample (for examining the relationship between sweet pattern and metabolic syndrome)

Then, PS was calculated based on a logistic model with covariates. Two groups (MetS and healthy group) were matched using 1:1 matching without replacement by the score. The distribution of primary variables in both groups prior to and after matching is presented in Table 2. Based on the PS, 506 individuals with MetS were matched with 506 healthy individuals. Due to lack of proper match, one of individuals was excluded from the study. After matching, the standard error of mean was decreased from 7.2 to 2.4% and this shows the quality of the matching ensures that the distribution of the variables was similar in the two groups studied (Fig. 1).

**Table 2:** Characteristics of adolescents with and without metabolic syndrome before and after matching

Variables	Before propensity score-matched			After propensity score-matched		
	Control group (n = 3020) No. (%)	Metabolic syndrome group (n = 735) No. (%)	P-value	Control group (n = 506) No. (%)	Metabolic syndrome group(n = 506) No. (%)	P-value
Age (years)						
Mean (SD)	12.42 (3.036)	12.55 (3.04)	0.29	12.59 (3.05)	12.67 (3.02)	0.70^a^
Age category (years)						
7–10	920 (30.50)	201 (27.35)	0.24	141 (27.87)	128 (25.30)	0.49
11–14	1293 (42.81)	327 (44.49)		211 (41.70)	229 (45.26)	
15–18	806 (26.69)	207 (28.16)		154 (30.43)	149 (29.45)	
Sex						
Boy	1584 (52.5)	390 (53.1)	0.77	277 (54.74)	280 (55.34)	0.85
Girl	1435 (47.5)	345 (46.9)		229 (45.26)	226 (44.66)	
Living area						
Urban	2167 (71.8)	565 (76.9)	0.005	380 (75.10)	387 (76.48)	0.61
Rural	853 (28.2)	170 (23.1)		126 (24.90)	119 (23.52)	
Father’s occupation						
Father died	65 (2.2)	20 (2.7)	0.38	11 (2.17)	12 (2.37)	0.61
Unemployed	199 (6.6)	45 (6.2)		28 (5.53)	31 (6.13)	
Workman/labor	741 (24.7)	162 (22.2)		105 (20.75)	117 (23.12)	
Employed/office work	623 (20.8)	155 (21.2)		130 (25.69)	106 (20.95)	
Agriculturist	397 (13.2)	87 (11.9)		57 (11.26)	63 (12.45)	
Self-employed	975 (32.5)	261 (35.8)		175 (34.58)	177 (34.98)	
Mother’s occupation						
Mother died	14 (0.5)	4 (0.5)	0.06	3 (0.59)	2 (0.40)	0.73
Housewife	2622 (87.1)	609 (83.2)		418 (82.61)	425 (83.99)	
Workman/labor	44 (1.5)	8 (1.1)		5 (0.99)	4 (0.79)	
Employed/office work	227 (7.5)	74 (10.1)		53 (10.47)	49 (9.68)	
Agriculturist	53 (1.8)	17 (2.3)		9 (1.78)	14 (2.77)	
Other	52 (1.7)	20 (2.7)		18 (3.56)	12 (2.37)	
Mother’s education (years)						
Mother died	11 (0.4)	3 (0.4)	0.02	3 (0.59)	1 (0.20)	0.54
< 6	1319 (43.7)	296 (40.5)		186 (36.76)	200 (39.53)	
6–9	617 (20.4)	129 (17.6)		108 (21.34)	91 (17.98)	
9–12	751 (24.9)	199 (27.2)		136 (26.88)	142 (28.06)	
> 12	320 (10.6)	104 (14.2)		73 (14.43)	72 (14.23)	
Possessing personal computer						
No	1599 (53.1)	364 (49.8)	0.11	231 (45.65)	242 (47.83)	0.49
Yes	1411 (46.9)	367 (50.2)		275 (54.35)	264 (52.17)	
School type						
Public	2736 (91)	676 (92.2)	0.30	476 (94.07)	467 (92.29)	0.26
Private	270 (9)	57 (7.8)		30 (5.93)	39 (7.71)	
Birth weight						
< 2500	305 (10.2)	62 (8.6)	0.008	43 (8.50)	42 (8.30)	0.79
2500–4000	2256 (75.8)	529 (73)		367 (72.53)	366 (72.33)	
> 4000	194 (6.5)	70 (9.7)		47 (9.29)	55 (10.87)	
Unknown	222 (7.5)	64 (8.8)		49 (9.68)	43 (8.50)	
Family history						
HTN						
No	1081 (36.9)	241 (33.4)	0.08	159 (31.42)	166 (32.81)	0.64
Yes	1848 (63.1)	480 (66.6)		347 (68.58)	340 (67.19)	
Hyperlipidemia						
No	1477 (49.5)	345 (47.5)	0.34	213 (42.09)	229 (45.26)	0.31
Yes	1507 (50.5)	381 (52.5)		293 (57.91)	277 (54.74)	
Diabetes						
No	1394 (46.5)	335 (45.9)	0.77	214 (42.29)	210 (41.50)	0.80
Yes	1605 (53.3)	395 (54.1)		292 (57.71)	296 (58.50)	
Obesity						
No	1565 (52.1)	354 (48.3)	0.06	240 (47.43)	233 (46.05)	0.66
Yes	1438 (47.9)	379 (51.7)		266 (52.57)	273 (53.95)	
Physical activity						
Low	955 (33.8)	229 (32.9)	0.11	158 (31.23)	138 (27.27)	0.09
Medium	913 (32.3)	253 (36.4)		166 (32.81)	199 (39.33)	
High	955 (33.8)	214 (30.7)		182 (35.97)	169 (33.40)	
Sleep duration						
Mean(SD)	8.58 (1.22)	8.49 (1.20)	0.07	8.49 (1.21)	8.50 (1.20)	0.90^a^
Parental BMI						
Mean (SD)	26.3 (4.96)	26.71 (4.86)	0.03	26.92 (4.96)	26.84 (4.88)	0.79^a^
Parent perception about students’ body image?						
Very thin	363 (12.09)	59 (8.10)	< 0.001	47 (9.29)	42 (8.30)	0.48
Slightly thin	801 (26.67)	156 (21.43)		98 (19.37)	113 (22.33)	
Normal	1438 (47.89)	343 (47.12)		256 (50.59)	235 (46.44)	
Slightly obese	352 (11.72)	145 (19.92)		92 (18.18)	97 (19.17)	
Very obese	49 (1.63)	25 (3.43)		13 (2.57)	19 (3.75)	
Student satisfaction about weight?						
Satisfy	1645 (54.49)	381 (51.84)	0.27	246 (48.62)	241 (47.63)	0.94
Somewhat satisfied	891 (29.51)	239 (32.52)		176 (34.78)	181 (35.77)	
Dissatisfied	483 (16)	115 (15.65)		84 (16.60)	84 (16.60)	
Watching TV						
Low	1436 (47.61)	321 (43.67)	0.055	224 (44.27)	221 (43.68)	0.85
High	1580 (52.39)	414 (56.33)		282 (55.73)	285 (56.32)	
Working with computer						
Low	2698 (91.55)	656 (91.36)	0.87	457 (90.32)	456 (90.12)	0.92
High	249 (8.45)	62 (8.64)		49 (9.68)	50 (9.88)	

^a^ According to the T-test, other P-values are based on Chi square test

Results of conditional logistic regression show that students with Sweet dietary patterns were at higher risk for abdominal obesity (OR: 1.29; 95% CI 1.01–1.66), elevated BP (OR 1.35; 95% CI 1.01–1.81) and MetS (OR 1.33; 95% CI 1.02–1.74). The two other dietary patterns were not associated with MetS and its components (P-value > 0.05) (Table 3).

**Table 3:** The results of the Conditional logistic of Dietary patterns relationship with the metabolic syndrome and its components

Dependent variable	Number of matching	Standard error of mean %	Adjusted OR (95% CI)	P-value
Sweet pattern				
Metabolic syndrome	506	2.4	*1.33 (1.02–1.74)*	*0.03*
Low HDL	745	2	1.05 (0.85**–**1.29)	0.67
High BP	398	4.3	*1.35 (1.01–1.81)*	*0.04*
Abdominal obesity	524	2.2	*1.29 (1.01–1.66)*	*0.04*
High FBG	533	3.5	0.98 (0.76–1.27)	0.9
High TG	206	5.9	1.02 (0.67–1.54)	0.92
Healthy pattern				
Metabolic syndrome	506	2.4	0.94 (0.72–1.23)	0.64
Low HDL	745	2	0.88 (0.71–1.1)	0.25
High BP	398	4.3	1.20 (0.89–1.61)	0.22
Abdominal obesity	524	2.2	1.22 (0.95–1.56)	0.10
High FBG	533	3.5	0.98 (0.76–1.28)	0.89
Low HDL	206	5.9	1.20 (0.79–1.81)	0.37
Western pattern				
Metabolic syndrome	506	2.4	1.01 (0.78–1.30)	0.95
Low HDL	745	2	0.94 (0.76–1.15)	0.54
High BP	398	4.3	0.97 (0.72–1.30)	0.83
Abdominal obesity	524	2.2	0.84 (0.65–1.08)	0.17
High FBG	533	3.5	1.06 (0.82–1.37)	0.65
Low HDL	206	5.9	1.16 (0.76–1.78)	0.47

To assess each of the components of metabolic syndrome, the covariates and the WHR was controlled

Criterion for diagnosing Components of the metabolic syndrome was obtained based on the IDF (international diabetes federation): TG ≥ 150 mg/dl, HDL-C ≤ 40 mg/dl, WHR greater than 0.5, FBG ≥ 100 mg/dl, and SBP or DBP ≥ 90th percentile for age, sex and height

*WHR* waist to height ratio, *HDL* high-density lipoprotein (mg/dl), *TG* triglyceride(mg/dl), *FBG* fasting blood glucose (mg/dl), *BP* blood pressure (mmHg)

## Discussion

The findings of this study showed that following the sweet pattern (rich in cakes, cookies, pastries, biscuits, chocolate, soft drinks, packaged fruit juice, tea, cubed sugar and sugar-sweetened tea) enhances the risk of MetS, hypertension and abdominal obesity in children; however, no relationship was found between western and healthy dietary patterns and MetS and its components.

In some studies, there was a significant relationship between a healthy diet and the MetS or its components. For example, a prospective study (2016) on 1369 girls aged 9–10 years with a 10-year follow-up revealed that a healthy diet in adolescence can prevent the accumulation of cardiometabolic risk factors as adolescent girls who mostly consumed dairy products, fruits and non-starchy vegetables were less likely to be affected by cardiometabolic risk factors [37]. A cross-sectional study (2008) on 4811 Iranian children and adolescents aged 6–18 years indicated that healthy diet (rich in dairy products, fruits and vegetables) is associated with decreased chances of HW phenotype (hypertriglyceridemic waist) [38]. Another cross-sectional study (2009) was conducted in Australia on 1139 14-year-old Australian adolescents and discovered the relationship between healthy diet and reduced blood sugar levels [20]. In other cross-sectional study (2008) in Australia conducted on 764 adolescents aged 12–18 years homogenized in terms of age, sex and physical activity, dietary pattern rich in fruits, salads, cereals and fish was associated with lower diastolic blood pressure in adolescents (16 years or above) [21]. Furthermore, a review study in 2015 showed that there is a relationship between following the diet high in fiber, low-fat dairy, whole grains, unsaturated fatty acids and high-density diet and reduced risk of obesity [39].

In the current study, no relationship was observed between a healthy diet and MetS and its components. This may be justified since low-fat and high-fat dairy products as well as starchy and non-starchy vegetables were not assessed in the employed questionnaire. Hence, the possible protective effect of healthy dietary pattern may be altered. In addition, some research studies also suggested the relationship between higher consumption of whole grains and legumes and the reduced risk of diabetes [40, 41]. In the present study, there was no information available on the consumption of whole grains and legumes. This may have reduced the possible protective effects of a healthy diet on the incidence of MetS and its components.

According to the findings of this study, following the sweet pattern (rich in cakes, cookies, pastries, biscuits, chocolate, soft drinks, packaged fruit juice, tea, cubed sugar and sugar with tea) enhances the risk of MetS, hypertension and abdominal obesity.

In the past, snacks such as sandwiches, nuts, dried fruits and fruits used to be prepared at home; however, they are now replaced by high calorie snacks with low nutritional value, such as cookies, cakes, candy, chocolate, ice cream, desserts, soft drinks, sweet drinks, potato chips, popcorn [42, 43]. Taking these snacks may have a negative impact on energy balance, weight gain and metabolic disorders [44–47]. Increased consumption of margarine and sugary and tasty snacks (like chips and cheese puffs) is positively associated with insulin Resistance [48]. Since the snacks, which are mostly sweet snacks, provide about 38% of daily energy requirements of children, they can play a key role in the incidence of overweight and obesity in children [49].

A review study in 2006 showed that consumption of sugary drinks is correlated with increased risk of obesity and overweight in children and adolescents [50]. One more cross-sectional study carried out in 2016 on 873 13-year-old students in Malaysia suggested that taking sugar-sweetened beverages is associated with harmful cardio metabolic consequences (increased waist circumference, elevated triglycerides, elevated blood sugar and reduced HDL) [51]. Fructose is a monosaccharide which naturally exists in fruits, honey and some vegetables; however, fructose is mainly available in industrial and commercial products such as soft drinks and sweetened drinks [52, 53]. A meta-analysis investigation in 2014 by Kelishadi et al. examining 15 clinical trial studies showed that fructose consumption is associated with increased FBS, triglycerides and systolic blood pressure. Furthermore, it was reversely associated with HDL [54]. Johnson et al. in their review study investigated the role of fructose in the prevalence of hypertension, obesity, MetS, kidney and cardiovascular diseases and recommended an increase in serum uric acid levels induced by fructose intake as the primary mechanism of increase in cardio-renal diseases such as hypertension [55]. Similarly, a cross-sectional study was conducted by Ford et al. on 12–17 year-old adolescents in the US, revealing a significant positive correlation between serum uric acid levels and the prevalence of MetS and some of its components [56]. Of other mechanisms are to induce less satiety after consuming sugary drinks compared to solid foods, leading to increased calorie intake [57, 58].

On the relationship between western dietary patterns and increased risk of MetS or its components, some studied achieved inconsistent findings. For example, a cross-sectional study (2010) in Australia on 1139 14-year-old female adolescents proposed a significant relationship between western dietary pattern and the increased risk of MetS, cholesterol and increased waist circumference and BMI [20]. Another cross-sectional study (2012) on 5267 children in China found that western dietary pattern (high in red meat, eggs, refined grains) is associated with increased risk of obesity and increased levels of blood sugar, systolic blood pressure, triglycerides, and reduced levels of HDL [22]. Consistently, the results of a case–control study matched based on the PS on 2984 Chinese adults in 2016 suggested a significant relationship between a diet rich in food proteins and fats and increased risk of MetS [16]. Another cross-sectional study on 486 40–60-year-old female teachers in Tehran showed that following the western dietary pattern (containing high amounts of refined grains, red meat, butter, processed meats and high-fat dairy) is associated with an increased risk of MetS [28]. A prospective study (2008) after 9 years of follow-up on 9514 adults showed the relationship between western dietary pattern and an increased incidence of MetS in adults; however, dairy consumption was followed by protective consequences [18]. A prospective study with 12 years of follow-up on the 42,504 40–75-year-old men put forth that the western dietary pattern (high in red meat, refined grains, high-fat dairy products, pastries, eggs, mayonnaise, butter, cheese and margarine) was associated with an increased risk of diabetes [19]. However, a nested case–control study on 698 subjects with a mean age of 43.6 years showed no significant relationship between western dietary and healthy patterns and the risk of type II diabetes [29].

The findings of the current study showed no significant relationship between the western dietary pattern (salty snacks, beer, fast food and coffee) and the risk of MetS and its components. Possible reasons may be as follows: Individuals with obesity or metabolic risk factors may follow a special diet or avoid consuming fast foods and salty snacks. This interrupts the correlation. This may be justified due to under-reporting of diet by people with metabolic diseases. Moreover, red meat in most studies largely contributed to determining the western pattern score. However, no information was in access regarding red meat in this study and this may have skewed the findings. Other probable reasons for inconsistencies in results include the difference in the dietary groups and the composition of foods comprising a dietary pattern, different factor loadings for each dietary group in the formation of a dietary pattern, and differences in variables controlled as intervening factors as well as social and cultural differences in response to a specific pattern so that the relationship between dietary patterns and metabolic risk factors in various studies may vary in different countries with regard to social, cultural and genetic differences [59, 60]. Another possible reason could be the lack of an accepted international MetS standard as different criteria were used in various studies.

The main limitation of this study was cross-sectional nature of the data, which prevented us reaching causal conclusions. Furthermore, a self-report tool was used to collect data on nutrition and physical activity; therefore, there was the possibility of information bias and reminisce. Additionally, although the FFQ questionnaire is more appropriate to collect nutritional data, the FFQ was not used in this study to identify dietary patterns and only 16 dietary groups were examined and no information is available on other food products such as meat, nuts and cereals, and beans. If the data on food items was collected quantitatively, individuals’ real intake would better be specified.

Furthermore, confounding variables such as parental income, psychological factors, puberty status of students and their eating behavior, such as food time, which are known as influential factors in MetS, are not controlled.

## Strengths

One of the strengths of this study enjoyed a large sample size like a national project and the results can be generalized to the entire population. Also, the standardized World Health Organization questionnaire was used in this study. Matching method based on the PS was adopted in this study, controlling a large number of primary and intervening variables. Finally, high quality of the data collected was further strength of the current study.

## Conclusion

Considering the above-mentioned findings, it can be claimed that following a sweet dietary pattern in children and adolescents increase the risk of MetS, abdominal obesity and hypertension in children and adolescents. Thus, metabolic risk factors may be prevented by enhancing families’ awareness in this regard and reducing the consumption of snacks with low nutritional value (especially sugary snacks).
